# Comparing the antibacterial activity of gaseous ozone and chlorhexidine solution on a tooth cavity model

**DOI:** 10.4317/jced.51130

**Published:** 2013-07-01

**Authors:** Arife Kapdan, Nurhan Öztaş, Zeynep Sümer

**Affiliations:** 1 DDS, PhD. Assistant Professor, Department of Pediatric Dentistry, Faculty of Dentistry, Cumhuriyet University, Sivas, Turkey; 2 DDS, PhD. Professor, Department of Pediatric Dentistry, Faculty of Dentistry, Gazi University, Ankara, Turkey; 3 DDS, PhD. Professor, Department of Microbiology, Faculty of Medicine, Cumhuriyet University, Sivas, Turkey

## Abstract

Objective: To evaluate the antibacterial activity of gaseous ozone and chlorhexidine solution on a tooth cavity model. Study Design: Twenty-one human molars were divided into 3 groups. Cavities were then cut into the teeth (4 per tooth, 28 cavities per group). After sterilization, the teeth were left in broth cultures of 106 colony-forming units (CFU) ml-1 of Streptococcus mutans (S. mutans) at 36°C for 48 h. The appropriate treatment followed (group A, control; group B, 2% chlorhexidine solution; and group C, 80s of treatment with ozone, and the cavities were then filled with composite resin. After 72h, the restorations were removed, dentin chips were collected with an excavator, and the total number of microorganisms was determined. Results: Both of the treatments significantly reduced the number of S. mutans present compared with the control group and there was a significant difference between the all groups in terms of the amount of the microorganisms grown (p < 0.05). Group B was beter than group C; and group C was better than group A. Moreover, it was found that the amount of the growth in the group of chlorhexidine was significantly less than that of the ozone group (p < 0.05). Conclusion: Chlorhexidine solution was the antibacterial treatment most efficacious on S. mutans; however, ozone application could be an anlternative cavity disinfection method because of ozone’s cavity disinfection activity.

** Key words:**Antibacterial activity, chlorhexidine, ozone, streptococcus mutans, tooth cavity.

## Introduction

In 1993, Anderson et al (1) described the traditional treatment of dental caries as the surgical removal of the diseased parts of the tooth structure and obturation of the area with an inert filling material. Thus, until recently, the primary way that dentists treated the clinical signs of caries infection was by the removal of diseased tissues. The goal of restorative dental treatment is to preserve tooth integrity for a maximum period of time (2). Bacteria remaining underneath restorations are regarded as one reason for secondary caries—and, thus, restoration failures (3). Furthermore, the presence of bacteria in dentin and their proximity to the pulp has clearly been associated with pulpal inflamation. During preparation, complete caries excavation based on clinical judgment (i.e., the color and texture of dentin in the cavity preparation) does not provide certainty as to whether bacteria remain. Caries bacteria present in dentinal tubules subjacent to deep dentinal lesions can be recovered from the nonexposed pulp tissue in the majority of cases (4). Because of this, pretreatment of the tooth surface with an antibacterial agent is useful in eliminating the harmful effects caused by either the residual bacteria or by bacterial microleakage (5).

Researchers have applied various alternative approaches to eliminate residual bacteria left in cavity preparations. Treatments with disinfectant washes and different antibacterial agents have been tested (6). Commercially available disinfectants containing compounds such as chlorhexidine (CHX) digluconate, disodium ethylenediaminetetraacetic acid (EDTA) dihydrate, sodium hypochlorite, hydrogen peroxide, and iodine are used to remove bacterial contaminants (7). Of these, CHX is commonly used to remove bacterial contaminants; it has a broad spectrum of action against both Gram-positive and Gram-negative microbes, although it is less effective with the latter (8). CHX is also effective in reducing the levels of* S. mutans* found on exposed carious root surfaces (9). Because of its antibacterial action, chlorhexidine application to the cavity prior to placement of the restoration had been recommended (10). In recent years, ozone gas therapy has been suggested as an alternative noninvasive treatment aiming to reduce the levels of caries-associated microorganisms. This form of therapy may therefore be an alternative or complementary treatment strategy in dentistry. Ozone is an energy-rich, highly unstable form of oxygen. It is a strong, fast oxidizer of cell walls and cytoplasmatic membranes of bacteria and is considered to be one of the best bactericidal, antiviral, and antifungal agents (11). The antibacterial effect of ozone on *S. mutans* has been evaluated (12,13).

One of the major environmental advantages of ozone is its low cytotoxicity, which, in clinical situations, may be due to the rapid degradation of ozone just after contact with organic compounds (14). Several studies have determined the antibacterial activity of dentin-bonding systems, conventional cements, or restorative materials using different methodologies (15). In most cases, simple direct inhibition tests, such as agar disc-diffusion methods, were used. Some studies (16,17) used in vitro tooth models to evaluate the antibacterial activity of bonding agents. There are few reports in the literature regarding the antibacterial effect of ozone on root and fissure carious lesions and effect of ozone on microorganisms that remain underneath restorations after excavation of caries (12,13). A tooth cavity model using clinically relevant in vitro conditions could be useful in further studies (18). The aim of the present study was to evaluate the antibacterial activity of gaseous ozone and chlorhexidine solution on a tooth cavity model.

## Material and Methods

- The ozone generator

The ozone generator KaVo HealozoneTM 2130C (KaVo Dental, Biberach, Germany) was used in this study. The device delivered gas with a flow of 615 ml/min. According to the manufacturer, the ozone concentration of the gas was 2100 ppm, +10 % (i.e., the device delivered approximately 65 µmol ozone/min).

- Microorganisms

*S. mutans* DSM 20523 was cultured overnight on Columbia Blood Agar (BD-Nr.279230) (Merck KGaA, Darmstadt, Germany) at 36°C (± 1°C) under an atmosphere of 7–8 % CO2. The microorganisms were harvested from the agar plate and diluted into 2ml of a peptone-yeast-bouillon (PYB) medium in order to produce a viable count of 106 colony-forming units (CFU) ml-1 of *S. mutans*, which was used for the experiments.

-Tooth cavity model

Twenty-one freshly extracted human non-carious third molars were used. The criteria for tooth selection included (1) intact crown enamel and (2) no caries or cracks. Teeth were examined using a stereomicroscope (Nikon SMZ 800, Nikon Corporation, Tokyo, Japan) for diagnose as sound. The teeth were cleaned with a toothbrush and water for 25 s each and then stored in sterile physiological saline (SPS). They were randomly divided into 3 groups of 7 teeth each. The groups tested were as follows: group A, control group without treatment; group B, 2 % chlorhexidine solution (Cavity Cleanser, Bisco, USA) application for 30 s; and group C, ozone application for 80 s. The enamel was removed from the occlusal part of the teeth to obtain flat dentinal surfaces by using a low-speed diamond saw (Isomet, Buehler Ltd, Lake Bluff, IL, USA). Four cylindrical cavities were prepared (diameter 2 mm, depth 2 mm) in the flat surface of each tooth without causing pulp exposure. Additionally, the roots of the teeth were removed using a diamond bur.

The teeth were sterilized by autoclaving for 15 min at 121 °C. The 4 cavities of each tooth were then dried with sterile paper points and every cavity was filled with 10 µl of 106 CFU ml-1 *S. mutans* suspension. The teeth were left in this condition for 3min so that the microorganisms could penetrate into the dentin. Next, each tooth was immersed in a bottle containing 5 ml of PYB medium, 50 µl of 106 CFU ml-1 *S. mutans*, and 1 % sucrose, then incubated at 36 °C for 48 h in order to establish an infected cavity.

Following incubation, the teeth were taken out of the bottles and the cavities were dried again with sterile paper points. Three of the cavities in each tooth were used as experimental cavities and one as a control for the dentin infection. From this control cavity, dentin chips were collected using different sizes of excavators (Asa Dental, 1709-32L, 1709-33L). The dentin chips were weighed for to standardize and then diluted 1:100 in PYB medium. The solution was stirred for 30 s, and a series of 10-fold dilutions was prepared. The numbers of *S. mutans* (CFU) were determined by viable plate counting on Columbia Blood Agar (17). Only teeth with control cavities that were infected with *S. mutans* at a degree higher than 105 CFU g-1 were counted in the study.

The other 3 cavities of each tooth were treated according to the group to which they belonged: the cavities of the control group (group A) were untreated, Cavity Cleanser was applied to the cavities of group B according to manufacturer’s instructions, and the teeth from group C were treated for 80 s with ozone. After the appropriate treatment, each cavity was filled with a piece of a sterile blue sponge (VDW, Munich, Germany) and a green composite resin (Twinky Star, Voco, Germany), which was polymerized for 20 s (Hilux Benlioğlu Dental, Turkey) without bonding agent. The teeth were kept separately in SPS at 36 °C for 72 h. The composite fillings were removed using different sterile diamond burs, which were first placed in a freezer for cooling, without coming into contact with the dentin walls of the cavity. Then the sponge was removed using sterile tweezers. The standardized amounts of dentin chips were collected with an excavator from the bottom and sides of each cavity and placed into sterile bottles. The dentin chips were weighed, and the numbers of *S. mutans* (CFU) recovered were determined.

- Statistical analysis

There were three independent groups and each group consisted of 21 individual cavities. The data analysis was done on SPSS for Windows ver. 11.5 pack software. Descriptive statistics were shown as a geometric mean (± standard deviation) for the number of microorganisms grown. The significance of the difference between the groups with regard to the number of the microorganisms grown was assessed with one-way variance analysis. If the results of the analysis of variance were found to be significant, the groups contributing the significant difference were determined using a post-hoc Tukey test. The level of significance was set at p < 0.05.

## Results

After the inoculated plates were incubated for 24 h, the number of the microorganisms that had grown on the plates was counted.  Table 1 shows the logarithmic values and standard deviation of the means in CFU/ml of the number of the microorganisms isolated from the cavities after applying materials.

**Table 1:**
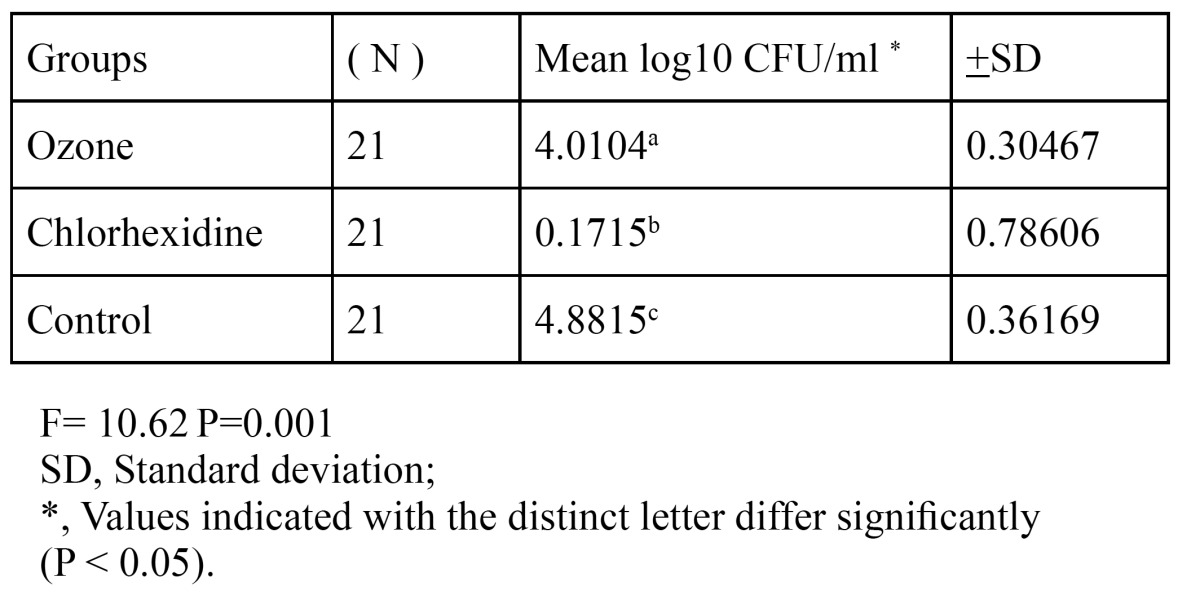
Logarithmic values of the means of the number of microorganisms isolated from the cavities.

There was a significant difference between the groups in terms of the amount of the microorganisms grown (p < 0.05). The decrease that was found in the groups of ozone and chlorhexidine in comparison with the control group was statistically significant (p < 0.05). Moreover, it was found that the amount of the growth in the group of chlorhexidine was significantly less than that of the ozone group (p < 0.05). The groups were ranked as chlorhexidine > ozone > control in decreasing order of the disinfectant quality.

## Discussion

In previous studies, it has been reported that the efficacy of ozone may be altered due to certain factors such as its concentration, time of exposure, ambient temperature, bacterial species, and organization; although it is an efficacious agent, further in vivo and in vitro studies are needed (19,20). Nogales et al (19) highlighted the need for further studies about the accurate time of application of ozone with respect to the depth of decay.

In the present in vitro study conducted, the time of ozone exposure on the teeth in the treatment group was decided to be 80 s, which is the longest time recommended in the previous studies on this issue, considering the fact that antibacterial effect is augmented by exposure time and concentration (18,21).

In a in vivo study, Baysan and Lynch (22) measured the ozone level within the air that leaked out from the silicone vacuum cups suctioning the tooth during ozone application for the treatment of early superficial root decays, and they reported that the measured level of ozone was within the range determined by the U.S. Food and Drug Administration (FDA) and European Union.

Several methods are used to test the properties of antibacterial restorative materials in vitro, including dentin bonding systems and cavity disinfectants. The most commonly used test in the studies is the agar diffusion test (17,23,24).

When the studies performed with agar diffusion method were reviewed, it was found that substrate pH, dentin thickness, diffusion capacity of the test material into agar and dentin, and incubation period might affect the results. Moreover, materials not releasing any antibacterial agent after polymerization such as MDPB are not suitable for testing with this method (25).

Ozer et al (17) developed a novel method called a tooth cavity model in their research on this issue. They noted that this method permitted them to apply materials in a way similar to clinical practice procedures, and it was a reliable method to assess the antibacterial effects of dentin-bonding agents.

In the present study evaluating antibacterial effects of chlorhexidine and ozone, the tooth cavity model developed by Ozer et al (17) was used, assuming that ozone application after suctioning with the HealOzone system was suitable only with the use of this method. The use of teeth with their roots removed, according to this method, allowed better penetration of microorganisms into dentinal tubules from the pulpal surface.

One cavity of each tooth was used to verify the degree of infection. Our pilot studies showed better results when the dentin chips were collected using an excavator instead of a carbide bur. This difference could be explained by the rise in temperature caused by the use of a carbide bur. The small piece of sterile blue sponge used under the blue composite resin allowed the removal of the fillings without contact with the cavity walls. Additionally, the diamond burs used for removing the composite fillings were placed in a freezer at -25 °C for cooling in order to exclude any excessive build up of heat within the cavity (17,18).

In the present study, a composite resin was used for filling the cavities. During the pilot work, different composites and temporary filling materials were tested for their antibacterial effect on *S. mutans* using the agar disc-diffusion test. The composite resin used in the present study showed no antibacterial activity for *S. mutans*, in contrast with the zinc oxide–based temporary filling material used by Ozer et al (17).

In this study, both antibacterial methods elicited a significant decrease in the number of *S. mutans* in comparison with the control group. A statistically significant difference was found between the groups in terms of the number of the microorganisms that grew in each (p < 0.05). There was a statistically significant decrease in the number of microorganisms in the groups of ozone and chlorhexidine in comparison with the control group (p < 0.05). Furthermore, it was found that the decrease in the number of microorganisms in the group of chlorhexidine was statistically significant in comparison with the ozone group (p < 0.05).

Chlorhexidine 2 %, which is contained in Cavity Cleanser, has a broad spectrum of activity against Gram-positive and Gram-negative microorganisms, yeast and fungi, and facultative anaerobe and aerobe microorganisms. However, it is indicated that the microorganisms that are the mostsensitive to chlorhexidine are Gram-positive cocci, particularly *S. mutans* (26,27). In most of the studies with chlorhexidine, resembling the results of the present study, it was reported that chlorhexidine decreased significantly the number of *S. mutans* (28).

Ozone, another antibacterial agent of preference in the present study, is a powerful bactericidal, antiviral, and antifungal agent; it rapidly oxidizes bacterial cytoplasmic membranes and cell walls (11). There are limited number of studies assessing the antibacterial effect of ozone on *S. mutans* (12,13). As a result of these studies, it was found that *S. mutans* is sensitive to ozone (29).

Baysan et al (12) showed that ozonated water was capable of significantly reducing *S. mutans* and *S. sobrinus* on saliva-coated glass beads when applied for 10 s. Furthermore, ozone reduced the number of microorganisms in more than 99 % after 10 s and 20 s application periods in root caries.

On the other hand, Baysan and Beighton (30) ascertained the effects of 40 s of ozone (or regular air) treatment on the number of bacteria invading the demineralized dentin of removed teeth with occlusal caries and reported that its effect on dentin was weak.

As a result of present study, it was determined that 80 s of ozone treatment with the purpose of cavity disinfection was effective on *S. mutans*, but not as much as reported by Baysan et al (12) who used ozonated water. That might be due to the fact that our study was performed on dentin surface, and for killing microorganisms penetrated into dentin tubules, ozone is not as effective as it is on the enamel surface. This result was consistent with the study conducted by Baysan and Beighton (30).

In the unique study about this issue relating to tooth cavity techniques, the antibacterial effects of ozone exposure method (40 s and 80 s) and 2 antibacterial bonding agents (Clearfill SE Bond, Clearfil Protect Bond) on *S. mutans* were compared. It was emphasized that both bonding systems and 80 s ozone exposure had more antibacterial efficacy than 40 s of ozone exposure did (18). Our study’s findings parallel the results of this study.

## Conclusions

It can be concluded from the present study that the chemical cavity disinfectant (Cavity Cleanser) was the antibacterial treatment most efficacious on *S. mutans*; however, ozone exposure could be also an efficient disinfectant when it is used appropriate concentration and period of time.
